# 3-(4-Chloro­phenyl­sulfin­yl)-2,4,6-trimethyl-1-benzofuran

**DOI:** 10.1107/S1600536810038419

**Published:** 2010-10-02

**Authors:** Hong Dae Choi, Pil Ja Seo, Byeng Wha Son, Uk Lee

**Affiliations:** a Department of Chemistry, Dongeui University, San 24 Kaya-dong Busanjin-gu, Busan 614-714, Republic of Korea; b Department of Chemistry, Pukyong National University, 599-1 Daeyeon 3-dong, Nam-gu, Busan 608-737, Republic of Korea

## Abstract

In the title mol­ecule, C_17_H_15_ClO_2_S, the 4-chloro­phenyl ring is oriented approximately perpendicular to the mean plane of the benzofuran ring [dihedral angle = 88.98 (4)°]. In the crystal, mol­ecules are linked through weak inter­molecular C—H⋯O and C—H⋯π inter­actions, forming right-hand pseudo-helices along the *a* axis.

## Related literature

For the structures of related benzofuran derivatives, see: Choi *et al.* (2010*a* ▶,*b* ▶). For the biological activity of benzofuran compounds, see: Aslam *et al.* (2006 ▶); Galal *et al.* (2009 ▶); Khan *et al.* (2005 ▶). For natural products containing benzofuran rings, see: Akgul & Anil (2003 ▶); Soekamto *et al.* (2003 ▶).
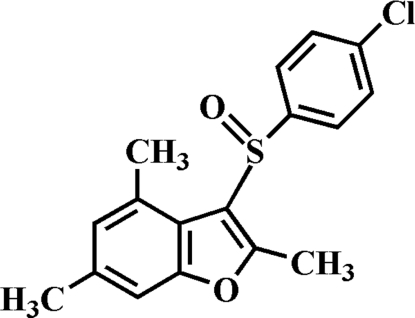


## Experimental

### 

#### Crystal data

C_17_H_15_ClO_2_S
*M* *_r_* = 318.80
Orthorhombic, 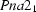

*a* = 12.1259 (10) Å
*b* = 19.3925 (16) Å
*c* = 6.4744 (5) Å
*V* = 1522.5 (2) Å^3^
*Z* = 4
Mo *K*α radiation
μ = 0.39 mm^−1^
*T* = 296 K
0.44 × 0.28 × 0.17 mm

#### Data collection

Bruker SMART APEXII CCD diffractometer
Absorption correction: multi-scan (*SADABS*; Bruker, 2009 ▶) *T*_min_ = 0.848, *T*_max_ = 0.937
14409 measured reflections
3468 independent reflections
3134 reflections with *I* > 2σ(*I*)
*R*_int_ = 0.028

#### Refinement

*R*[*F* ^2^ > 2σ(*F* ^2^)] = 0.033
*wR*(*F* ^2^) = 0.089
*S* = 1.03
3468 reflections
194 parameters
1 restraint
H-atom parameters constrained
Δρ_max_ = 0.16 e Å^−3^
Δρ_min_ = −0.24 e Å^−3^
Absolute structure: Flack (1983 ▶), 1561 Friedel pairs
Flack parameter: −0.03 (6)

### 

Data collection: *APEX2* (Bruker, 2009 ▶); cell refinement: *SAINT* (Bruker, 2009 ▶); data reduction: *SAINT*; program(s) used to solve structure: *SHELXS97* (Sheldrick, 2008 ▶); program(s) used to refine structure: *SHELXL97* (Sheldrick, 2008 ▶); molecular graphics: *ORTEP-3* (Farrugia, 1997 ▶) and *DIAMOND* (Brandenburg, 1998 ▶); software used to prepare material for publication: *SHELXL97*.

## Supplementary Material

Crystal structure: contains datablocks global, I. DOI: 10.1107/S1600536810038419/pv2330sup1.cif

Structure factors: contains datablocks I. DOI: 10.1107/S1600536810038419/pv2330Isup2.hkl

Additional supplementary materials:  crystallographic information; 3D view; checkCIF report

## Figures and Tables

**Table 1:** Hydrogen-bond geometry (Å, °) *Cg* is the centroid of the C12–C17 4-chloro­phenyl ring.

*D*—H⋯*A*	*D*—H	H⋯*A*	*D*⋯*A*	*D*—H⋯*A*
C13—H13⋯O2^i^	0.93	2.62	3.471 (2)	152
C11—H11*C*⋯*Cg*^ii^	0.96	2.85	3.667 (2)	144

Symmetry codes: (i) 
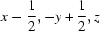
; (ii) 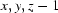.

**Fig. 1.**
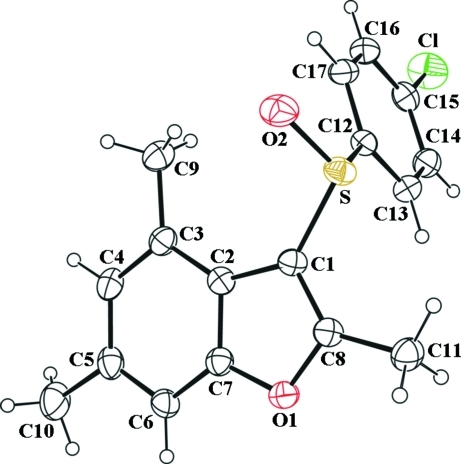
The molecular structure of the title compound with the atom numbering scheme. Displacement ellipsoids are drawn at the 30% probability level. H atoms are presented as spheres of arbitrary radii.

**Fig. 2.**
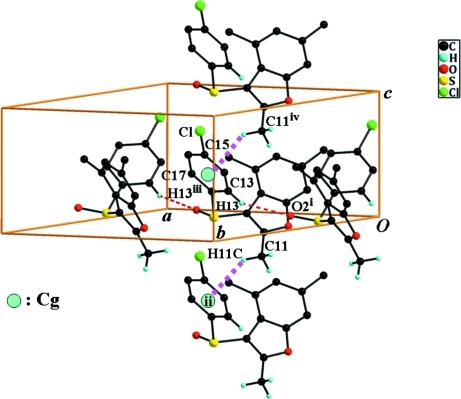
A view of the C–H···O and C–H···π interactions (dotted lines) in the crystal structure of the title compound; Cg denotes the centroid of the C12-C17 ring. Hydrogen atoms not involved in hydrogen bonds have been excluded for clarity. Symmetry codes: (i) x -1/2, - y + 1/2, z (ii) x, y, z - 1 (iii) x 1/2, - y + 1/2, z (iv) x, y, z + 1

## supplementary crystallographic information

### Comment

Many compounds involving a benzofuran ring system exhibit important pharmacological properties such as antifungal, antimicrobial, antitumor and antiviral activities (Aslam *et al.*, 2006; Galal *et al.*, 2009; Khan *et al.*, 2005). These compounds widely occur in nature (Akgul & Anil, 2003; Soekamto *et al.*, 2003). As a part of our study of the substituent effect on the solid state structures of 3-(4-chlorophenylsulfinyl)-2-methyl-1-benzofuran analogues (Choi *et al.*, 2010**a*,b*), we report herein the crystal structure of the title compound.

In the title molecule (Fig. 1), the benzofuran unit is essentially planar, with a mean deviation of 0.009 (2) Å from the least-squares plane defined by the nine constituent atoms. The 4-chlorophenyl ring makes a dihedral angle of 88.98 (4)° with the mean plane of the benzofuran fragment. The crystal packing (Fig. 2) is stabilized by a weak intermolecular C–H···O hydrogen bond between the 4-chlorophenyl H atom and the oxygen of the S═O unit (C13–H13···O2^i^; Table 1), and by an intermolecular C–H···π interaction between a methyl H atom and the 4-chlorophenyl ring (C11-H11C···Cg^ii^; Table 1, Cg is the centroid of the C12–C17 4-chlorophenyl ring).

The title compound is crystallized in the non-centrosymmetric space group *Pna*2_1_ in spite of having no asymmetric C atoms. The space group is caused by a right hand pseudo-helix along the *a* axis (Fig. 2).

### Experimental

3-Chloroperoxybenzoic acid (77%, 291 mg, 1.3 mmol) was added in small portions to a stirred solution of 3-(4-chlorophenylsulfanyl)-2,4,6-trimethyl-1-benzofuran (363 mg, 1.2 mmol) in dichloromethane (30 mL) at 273 K. After being stirred at room temperature for 3h, the mixture was washed with saturated sodium bicarbonate solution and the organic layer was separated, dried over magnesium sulfate, filtered and concentrated at reduced pressure. The residue was purified by column chromatography (silica gel, hexane–ethyl acetate, 2:1 v/v) to afford the title compound as a colorless solid [yield 83%, m.p. 452–453 K; *R*_f_ = 0.62 (hexane–ethyl acetate, 2:1 v/v)]. Single crystals suitable for X-ray diffraction were prepared by slow evaporation of a solution of the title compound in ethyl acetate at room temperature.

### Refinement

All H atoms were positioned geometrically and refined using a riding model, with C–H = 0.93 Å for aryl and 0.96 Å for methyl H atoms. *U*_iso_(H) = 1.2*U*_eq_(C) for aryl and 1.5*U*_eq_(C) for methyl H atoms.

### Crystal data

**Table:**

C_17_H_15_ClO_2_S	*F*(000) = 664
*M**_r_* = 318.80	*D*_x_ = 1.391 Mg m^−^^3^
Orthorhombic, *P**n**a*2_1_	Mo *K*α radiation, λ = 0.71073 Å
Hall symbol: P 2c -2n	Cell parameters from 5206 reflections
*a* = 12.1259 (10) Å	θ = 2.7–26.9°
*b* = 19.3925 (16) Å	µ = 0.39 mm^−^^1^
*c* = 6.4744 (5) Å	*T* = 296 K
*V* = 1522.5 (2) Å^3^	Block, colourless
*Z* = 4	0.44 × 0.28 × 0.17 mm

### Data collection

**Table:**

Bruker SMART APEXII CCD diffractometer	3468 independent reflections
Radiation source: rotating anode	3134 reflections with *I* > 2σ(*I*)
graphite multilayer	*R*_int_ = 0.028
Detector resolution: 10.0 pixels mm^-1^	θ_max_ = 27.5°, θ_min_ = 2.0°
φ and ω scans	*h* = −14→15
Absorption correction: multi-scan (*SADABS*; Bruker, 2009)	*k* = −25→25
*T*_min_ = 0.848, *T*_max_ = 0.937	*l* = −8→8
14409 measured reflections	

### Refinement

**Table:**

Refinement on *F*^2^	Secondary atom site location: difference Fourier map
Least-squares matrix: full	Hydrogen site location: difference Fourier map
*R*[*F*^2^ > 2σ(*F*^2^)] = 0.033	H-atom parameters constrained
*wR*(*F*^2^) = 0.089	*w* = 1/[σ^2^(*F*_o_^2^) + (0.0507*P*)^2^ + 0.1747*P*] where *P* = (*F*_o_^2^ + 2*F*_c_^2^)/3
*S* = 1.03	(Δ/σ)_max_ < 0.001
3468 reflections	Δρ_max_ = 0.16 e Å^−^^3^
194 parameters	Δρ_min_ = −0.24 e Å^−^^3^
1 restraint	Absolute structure: Flack (1983), 1561 Friedel pairs
Primary atom site location: structure-invariant direct methods	Flack parameter: −0.03 (6)

### Special details

**Table:**

Geometry. All esds (except the esd in the dihedral angle between two l.s. planes) are estimated using the full covariance matrix. The cell esds are taken into account individually in the estimation of esds in distances, angles and torsion angles; correlations between esds in cell parameters are only used when they are defined by crystal symmetry. An approximate (isotropic) treatment of cell esds is used for estimating esds involving l.s. planes.
Refinement. Refinement of F^2^ against ALL reflections. The weighted R-factor wR and goodness of fit S are based on F^2^, conventional R-factors R are based on F, with F set to zero for negative F^2^. The threshold expression of F^2^ > 2sigma(F^2^) is used only for calculating R-factors(gt) etc. and is not relevant to the choice of reflections for refinement. R-factors based on F^2^ are statistically about twice as large as those based on F, and R- factors based on ALL data will be even larger.

### Fractional atomic coordinates and isotropic or equivalent isotropic displacement parameters (Å^2^)

**Table:**

	*x*	*y*	*z*	*U*_iso_*/*U*_eq_	
S	0.58821 (4)	0.24616 (2)	0.00652 (10)	0.04345 (12)	
Cl	0.49572 (6)	0.44910 (4)	0.73907 (14)	0.0840 (2)	
O1	0.32383 (10)	0.13450 (6)	−0.0716 (2)	0.0445 (3)	
O2	0.69629 (11)	0.21294 (7)	0.0504 (3)	0.0574 (4)	
C1	0.48058 (14)	0.18533 (8)	0.0312 (3)	0.0377 (4)	
C2	0.45571 (14)	0.13178 (8)	0.1815 (3)	0.0373 (4)	
C3	0.50175 (15)	0.10563 (9)	0.3645 (3)	0.0418 (4)	
C4	0.44487 (17)	0.05245 (10)	0.4608 (3)	0.0483 (5)	
H4	0.4739	0.0343	0.5821	0.058*	
C5	0.34541 (15)	0.02446 (10)	0.3847 (4)	0.0503 (5)	
C6	0.30164 (16)	0.04966 (9)	0.2031 (4)	0.0478 (5)	
H6	0.2369	0.0319	0.1473	0.057*	
C7	0.35838 (16)	0.10246 (9)	0.1083 (3)	0.0400 (4)	
C8	0.40014 (15)	0.18465 (9)	−0.1136 (3)	0.0405 (4)	
C9	0.60623 (18)	0.13287 (11)	0.4595 (3)	0.0535 (5)	
H9A	0.5897	0.1728	0.5416	0.080*	
H9B	0.6571	0.1453	0.3521	0.080*	
H9C	0.6386	0.0979	0.5452	0.080*	
C10	0.2873 (2)	−0.03139 (12)	0.5061 (6)	0.0758 (7)	
H10A	0.2113	−0.0334	0.4652	0.114*	
H10B	0.2919	−0.0212	0.6509	0.114*	
H10C	0.3219	−0.0750	0.4792	0.114*	
C11	0.37730 (19)	0.22734 (11)	−0.2970 (4)	0.0509 (5)	
H11A	0.3126	0.2547	−0.2729	0.076*	
H11B	0.3654	0.1980	−0.4144	0.076*	
H11C	0.4390	0.2571	−0.3232	0.076*	
C12	0.55633 (14)	0.30025 (8)	0.2240 (3)	0.0390 (4)	
C13	0.45411 (16)	0.33225 (10)	0.2400 (4)	0.0485 (5)	
H13	0.3986	0.3226	0.1453	0.058*	
C14	0.43614 (17)	0.37853 (10)	0.3984 (4)	0.0522 (5)	
H14	0.3684	0.4006	0.4111	0.063*	
C15	0.51924 (18)	0.39180 (9)	0.5371 (4)	0.0491 (5)	
C16	0.62023 (17)	0.36065 (9)	0.5241 (4)	0.0497 (4)	
H16	0.6748	0.3699	0.6211	0.060*	
C17	0.63960 (16)	0.31487 (10)	0.3627 (4)	0.0464 (5)	
H17	0.7084	0.2942	0.3483	0.056*	

### Atomic displacement parameters (Å^2^)

**Table:**

	*U*^11^	*U*^22^	*U*^33^	*U*^12^	*U*^13^	*U*^23^
S	0.0401 (2)	0.0491 (2)	0.0411 (2)	−0.00916 (18)	0.0045 (2)	0.0011 (2)
Cl	0.0895 (5)	0.0805 (4)	0.0819 (5)	0.0023 (4)	0.0111 (4)	−0.0341 (4)
O1	0.0401 (7)	0.0475 (7)	0.0461 (8)	−0.0058 (5)	−0.0063 (6)	0.0037 (6)
O2	0.0389 (7)	0.0638 (8)	0.0695 (11)	−0.0017 (6)	0.0088 (7)	−0.0075 (8)
C1	0.0372 (8)	0.0412 (8)	0.0347 (9)	−0.0036 (6)	0.0007 (8)	−0.0015 (7)
C2	0.0358 (9)	0.0383 (8)	0.0377 (9)	0.0011 (7)	0.0055 (8)	−0.0016 (7)
C3	0.0408 (9)	0.0443 (9)	0.0403 (10)	0.0061 (7)	0.0006 (8)	−0.0016 (8)
C4	0.0461 (10)	0.0503 (10)	0.0485 (12)	0.0094 (8)	0.0031 (9)	0.0120 (8)
C5	0.0449 (11)	0.0427 (9)	0.0632 (14)	0.0044 (7)	0.0116 (10)	0.0114 (9)
C6	0.0366 (9)	0.0418 (8)	0.0649 (14)	−0.0034 (7)	0.0024 (9)	0.0049 (9)
C7	0.0381 (9)	0.0405 (8)	0.0416 (10)	0.0022 (7)	0.0013 (8)	0.0001 (8)
C8	0.0407 (9)	0.0428 (9)	0.0380 (10)	−0.0026 (7)	0.0010 (8)	−0.0006 (7)
C9	0.0506 (12)	0.0615 (12)	0.0485 (14)	0.0018 (9)	−0.0076 (10)	0.0025 (9)
C10	0.0633 (14)	0.0679 (13)	0.096 (2)	−0.0044 (11)	0.0056 (16)	0.0395 (15)
C11	0.0522 (11)	0.0588 (11)	0.0416 (11)	−0.0017 (10)	−0.0031 (9)	0.0068 (9)
C12	0.0379 (9)	0.0373 (8)	0.0417 (10)	−0.0054 (7)	−0.0014 (8)	0.0037 (8)
C13	0.0431 (10)	0.0481 (10)	0.0542 (12)	−0.0012 (8)	−0.0102 (10)	0.0042 (9)
C14	0.0441 (10)	0.0460 (10)	0.0666 (14)	0.0061 (8)	−0.0001 (10)	0.0011 (10)
C15	0.0583 (12)	0.0382 (9)	0.0508 (12)	−0.0032 (8)	0.0049 (10)	−0.0034 (9)
C16	0.0481 (10)	0.0501 (10)	0.0509 (12)	−0.0060 (8)	−0.0088 (11)	−0.0027 (10)
C17	0.0358 (9)	0.0481 (9)	0.0553 (13)	−0.0030 (8)	−0.0046 (9)	−0.0018 (9)

### Geometric parameters (Å, °)

**Table:**

S—O2	1.4878 (15)	C9—H9A	0.9600
S—C1	1.7665 (17)	C9—H9B	0.9600
S—C12	1.798 (2)	C9—H9C	0.9600
Cl—C15	1.740 (2)	C10—H10A	0.9600
O1—C8	1.370 (2)	C10—H10B	0.9600
O1—C7	1.385 (2)	C10—H10C	0.9600
C1—C8	1.353 (3)	C11—H11A	0.9600
C1—C2	1.455 (2)	C11—H11B	0.9600
C2—C7	1.393 (3)	C11—H11C	0.9600
C2—C3	1.405 (3)	C12—C17	1.381 (3)
C3—C4	1.389 (3)	C12—C13	1.390 (3)
C3—C9	1.504 (3)	C13—C14	1.380 (3)
C4—C5	1.411 (3)	C13—H13	0.9300
C4—H4	0.9300	C14—C15	1.374 (3)
C5—C6	1.379 (3)	C14—H14	0.9300
C5—C10	1.512 (3)	C15—C16	1.368 (3)
C6—C7	1.378 (3)	C16—C17	1.391 (3)
C6—H6	0.9300	C16—H16	0.9300
C8—C11	1.474 (3)	C17—H17	0.9300
			
O2—S—C1	110.14 (8)	H9A—C9—H9C	109.5
O2—S—C12	107.01 (9)	H9B—C9—H9C	109.5
C1—S—C12	99.20 (8)	C5—C10—H10A	109.5
C8—O1—C7	106.32 (15)	C5—C10—H10B	109.5
C8—C1—C2	107.89 (15)	H10A—C10—H10B	109.5
C8—C1—S	118.45 (14)	C5—C10—H10C	109.5
C2—C1—S	133.66 (14)	H10A—C10—H10C	109.5
C7—C2—C3	118.46 (16)	H10B—C10—H10C	109.5
C7—C2—C1	103.84 (16)	C8—C11—H11A	109.5
C3—C2—C1	137.70 (17)	C8—C11—H11B	109.5
C4—C3—C2	116.71 (17)	H11A—C11—H11B	109.5
C4—C3—C9	119.73 (19)	C8—C11—H11C	109.5
C2—C3—C9	123.55 (18)	H11A—C11—H11C	109.5
C3—C4—C5	123.59 (19)	H11B—C11—H11C	109.5
C3—C4—H4	118.2	C17—C12—C13	120.78 (18)
C5—C4—H4	118.2	C17—C12—S	118.16 (14)
C6—C5—C4	119.37 (18)	C13—C12—S	120.72 (15)
C6—C5—C10	121.2 (2)	C14—C13—C12	119.13 (19)
C4—C5—C10	119.5 (2)	C14—C13—H13	120.4
C7—C6—C5	116.80 (19)	C12—C13—H13	120.4
C7—C6—H6	121.6	C15—C14—C13	119.45 (18)
C5—C6—H6	121.6	C15—C14—H14	120.3
C6—C7—O1	123.85 (18)	C13—C14—H14	120.3
C6—C7—C2	125.07 (18)	C16—C15—C14	122.23 (19)
O1—C7—C2	111.06 (15)	C16—C15—Cl	118.38 (18)
C1—C8—O1	110.88 (17)	C14—C15—Cl	119.39 (16)
C1—C8—C11	133.50 (17)	C15—C16—C17	118.6 (2)
O1—C8—C11	115.58 (16)	C15—C16—H16	120.7
C3—C9—H9A	109.5	C17—C16—H16	120.7
C3—C9—H9B	109.5	C12—C17—C16	119.75 (18)
H9A—C9—H9B	109.5	C12—C17—H17	120.1
C3—C9—H9C	109.5	C16—C17—H17	120.1
			
O2—S—C1—C8	−136.54 (15)	C1—C2—C7—C6	−178.86 (18)
C12—S—C1—C8	111.43 (16)	C3—C2—C7—O1	179.46 (15)
O2—S—C1—C2	43.6 (2)	C1—C2—C7—O1	−0.29 (19)
C12—S—C1—C2	−68.45 (18)	C2—C1—C8—O1	−0.3 (2)
C8—C1—C2—C7	0.3 (2)	S—C1—C8—O1	179.82 (13)
S—C1—C2—C7	−179.78 (15)	C2—C1—C8—C11	177.3 (2)
C8—C1—C2—C3	−179.3 (2)	S—C1—C8—C11	−2.6 (3)
S—C1—C2—C3	0.6 (3)	C7—O1—C8—C1	0.1 (2)
C7—C2—C3—C4	−0.9 (3)	C7—O1—C8—C11	−177.95 (16)
C1—C2—C3—C4	178.8 (2)	O2—S—C12—C17	14.13 (17)
C7—C2—C3—C9	−179.91 (18)	C1—S—C12—C17	128.60 (15)
C1—C2—C3—C9	−0.3 (3)	O2—S—C12—C13	−172.53 (15)
C2—C3—C4—C5	−0.1 (3)	C1—S—C12—C13	−58.05 (16)
C9—C3—C4—C5	178.97 (19)	C17—C12—C13—C14	−0.7 (3)
C3—C4—C5—C6	1.1 (3)	S—C12—C13—C14	−173.88 (15)
C3—C4—C5—C10	−177.4 (2)	C12—C13—C14—C15	−0.3 (3)
C4—C5—C6—C7	−1.1 (3)	C13—C14—C15—C16	0.1 (3)
C10—C5—C6—C7	177.4 (2)	C13—C14—C15—Cl	−178.98 (17)
C5—C6—C7—O1	−178.26 (17)	C14—C15—C16—C17	1.0 (3)
C5—C6—C7—C2	0.1 (3)	Cl—C15—C16—C17	−179.89 (16)
C8—O1—C7—C6	178.73 (19)	C13—C12—C17—C16	1.8 (3)
C8—O1—C7—C2	0.14 (19)	S—C12—C17—C16	175.18 (16)
C3—C2—C7—C6	0.9 (3)	C15—C16—C17—C12	−2.0 (3)

### Hydrogen-bond geometry (Å, °)

**Table:**

Cg is the centroid of the C12–C17 4-chlorophenyl ring.

**Table:**

*D*—H···*A*	*D*—H	H···*A*	*D*···*A*	*D*—H···*A*
C13—H13···O2^i^	0.93	2.62	3.471 (2)	152
C11—H11C···Cg^ii^	0.96	2.85	3.667 (2)	144

Symmetry codes: (i) *x*−1/2, −*y*+1/2, *z*; (ii) *x*, *y*, *z*−1.
